# Genetic drift evolution under vaccination pressure among H5N1 Egyptian isolates

**DOI:** 10.1186/1743-422X-8-283

**Published:** 2011-06-08

**Authors:** Ahmed S Abdel-Moneim, Manal A Afifi, Magdy F El-Kady

**Affiliations:** 1 Department of Virology, Faculty of Veterinary Medicine, Beni-Suef University, Beni-Suef, Egypt; 2 Department of Microbiology, Virology Division, College of Medicine, Taif University, Al-Taif, Saudi Arabia; 3 Department of Poultry Diseases, Faculty of Veterinary Medicine, Cairo University, Giza, Egypt; 4 Department of Poultry Diseases, Faculty of Veterinary Medicine, Beni-Suef University, Beni-Suef, Egypt

**Keywords:** H5N1, Egypt, drift, avian influenza

## Abstract

**Background**

The highly pathogenic H5N1 is a major avian pathogen that intensively affects the poultry industry in Egypt even in spite of the adoption of vaccination strategy. Antigenic drift is among the strategies the influenza virus uses to escape the immune system that might develop due to the pressure of extensive vaccination. H5N1 mutates in an intensified manner and is considered a potential candidate for the possible next pandemic with all the catastrophic consequences such an eventuality will entail.

**Methods**

H5N1 was isolated from the pooled organ samples of four different affected flocks in specific pathogen free embryonated chicken eggs (SPF-ECE). A reverse transcriptase polymerase chain reaction (RT-PCR) was performed to the haemagglutingin and neuraminidase. Sequencing of the full length haemagglutingin was performed. Sequence analyses of the isolated strains were performed and compared to all available H5N1 from Egyptian human and avian strains in the flu database. Changes in the different amino acid that may be related to virus virulence, receptor affinity and epitope configuration were assigned and matched with all available Egyptian strains in the flu database.

**Results**

One out of the four strains was found to be related to the B2 Egyptian lineage, 2 were related to A1 lineage and the 4^th ^was related to A2 lineage. Comparing data obtained from the current study by other available Egyptian H5N1 sequences remarkably demonstrates that amino acid changes in the immune escape variants are remarkably restricted to a limited number of locations on the HA molecule during antigenic drift. Molecular diversity in the HA gene, in relevance to different epitopes, were not found to follow a regular trend, suggesting abrupt cumulative sequence mutations. However a number of amino acids were found to be subjected to high mutation pressure.

**Conclusion**

The current data provides a comprehensive view of HA gene evolution among H5N1 subtype viruses in Egypt. Egyptian H5N1-AIVs are constantly undergoing genetic changes and reveal a complex pattern of drifts. These findings raise the concerns about the value of using influenza vaccines in correlation with the development of antigenic drift in influenza epidemics.

## Background

The highly pathogenic H5N1 influenza virus is currently panzootic in Egyptian poultry populations and crosses species barriers to humans and animals. H5N1 viruses are classified into 10 distinct initial clades (numbered 0-9) based on the HA gene [1]. H5N1 virus strains from European-Middle Eastern-African (EMA) origin were assigned to three clades (EMA-1-3) [2] and referred to as sub-clades 2.2.1.-2.2.3 [3]. Egyptian H5N1 viruses were exclusively classified as clade 2.2.1, which were further subdivided into two major groups A (A1-A5) and B (B1-B2) [4]. Following the widespread of HPAI H5N1 in Egypt, authorities began culling and vaccination to control the spread of the disease in poultry. H5N1 and several H5N2 inactivated vaccines were imported in a trial to control the H5N1 in Egypt. Nevertheless, it still endemic in the country and continues to constitute a major challenge to poultry population. The long-term pattern of endemic status increases the opportunity for the emergence of potential pandemic strains through further adaptation by genetic mutation or reassortment [5]. The HA gene is a target of neutralizing antibodies and a classic example of an antigenically drifting protein [6]where a large number of point mutations accumulate in the epitope regions or antibody combining regions [7]. Antigenic drift at the epitope regions represents the most important hindrance to vaccine development since an effective vaccination is possible only when the epidemic strain matches with the vaccine strain [8]. In additions, suboptimal vaccination strategies could result in enhanced antigenic drift accelerated by the immune pressure due to prior immunization exerted on the replicating viruses and continuous interspecies and intraspecies transmission [9]. However, it is generally accepted that the highly error-prone replication of influenza viruses and viral genome reassortment enhance the robustness of an efficient evolutionary capacity of the virus [10,11]

In the present paper, we isolated and characterized four H5N1 strains from Egyptian commercial chicken farms to get a deep insight into possible intra- and inter-subtype sequence variations. The genetic and phylogenetic properties of these viruses were determined and compared to each other and to all available Egyptian published sequences in the flu database to determine the vigor of the mutational events of HA. We tried to address the mutations change over time in HA in correlation to their functional impact.

## Materials and methods

### Virus isolation

Liver, spleen and proventriculus organ samples were collected from succumbed birds from four infected poultry farms (F7-F10) in three different Governorates (Fayoum, Qaluobiya and Giza). Birds in all farms received the H5N2 or H5N1 vaccine 2-3 weeks before developing the characteristic signs of the disease. Organs were routinely processed individually, infected materials were pooled for each farm, centrifuged at 500 × g for 10 min. and gentamicin sulfate solution (50 mg/ml) was added prior to inoculation into the allantoic cavity of five, 10-day-SPF-ECE (100 μl/egg). Inoculated embryos were incubated at 37°C for 24-48 hrs.

### Haemagglutination

The allantoic fluid of pooled infected embryos from each sample (F7-F10) was subjected to haemagglutination in 25-μl volume in 96-well HI plates. Two fold serial dilution of the infected allantoic fluid was performed for each sample. Equal volumes of 1% suspension of chicken erythrocytes were dispensed to each well [12]. Isolates possessed HA titers ≥ 3 log_2 _were suspected to be positive. The microtiter plate was left at room temperature until the negative control well exhibited a tight well circumscribed button of unagglutinated sedimented cells. The result of each sample was numerically reported.

### Avian influenza virus antigen detection

The allantoic fluid that showed haemagglutination was screened for AIV group and H5 antigens using the rapid chromatographic strip test (Animal Genetic Inc. Korea) according to manufacturer instructions.

### Viral RNA extraction and RT PCR

Viral RNA was extracted from virus containing the chorioallantoic membrane (CAM) homogenate of each sample using a SV Total RNA Isolation System (Promega Corporation, Madison, Wis.). RT-PCR amplification for the full length of both NA and HA genes of H5N1 were performed using the Verso™ 1 step RT PCR (Thermo Fisher Scientific Inc.). While a single set of primers flanking 1345 bp was used for NA[13], for HA, four sets of primers, flanking overlapping regions of the full length gene, were used (Table 1).

**Table 1:** Oligonucleotides used for amplification of the haemagglutinin and neuraminidase genes of H5N1

Name	Sense	Primer sequence (5'-3')	Product size	Reference
HA a	For	agcraaagcaggggt	699	
	Rev	ctctgrttyagtgttgatgt		FLI -Germany*
HA b	For	gagcagaataaaycattttgaga	801	
	Rev	tgagtggattctttgtctgcagc		
HA c	For	acatcaacactraaycagag	716	
	Rev	aagtctagagttctctcattyt		
HA d	For	gctgcagacaaagaatccactca	615	
	Rev	gaccagtagaaacaagggtgtttt		
NA	For	atgaatccaaatcagaag	1345	[13]
	Rev	tgtcaatggtgaatggcaac		

*Primer sequences for HA (a-d) were ordered according to information provided by Dr. Sasan R Fereidouni, the Laboratory of Molecular and Biological Characterization of AIV, Friedrich Loeffler Institute, Germany.

### Sequencing

RT-PCR amplicons were subjected to electrophoresis in 1% agarose gel. HA specific bands were excised and purified with the QIAquick gel extraction kit (Qiagen). Each purified RT-PCR products was sequenced directly in both forward and reverse directions (Macrogen, Korea). Sequences were trimmed to remove amplicon primer-linker sequence and assembled. All sequence data used in this study are available in the GenBank database (Accession No: JF357720-JF357723).

### Multiple sequence analysis and phylogenetic tree

Comparative analyses were performed using the CLUSTAL W multiple sequence alignment program, Mega 4.1 [14]. AIV representative sequences used for the alignments were obtained from the GenBank and EMBL databases. The phylogenetic tree was constructed by using the neighbour-joining method with Kimura two-parameter distances by using the Mega 4.1. The reliability of internal branches was assessed by 1000 bootstrap replications and the p-distance substitution model. N-linked potential glycosylation sites were analyzed with the NetNglyc server with predicted threshold values above 0.5[15].

### Deduced amino acid sequence analysis

We analyzed the HA deduced amino acid sequences of 4 isolated strains (F7-F10) and compared them with Egyptian H5N1 isolates available in the flu database. We used a complete collection of 315 H5N1 avian strains isolated from 2006 to 2010 in Egypt (2006 [n = 37], 2007 [n = 78], 2008 [n = 82], 2009 [n = 74], 2010[n = 44]), as well as 97 Egyptian human isolates, in order to screen the sites that had undergone amino acid intensive changes in recently determined different epitopes [16]. Amino acid residues associated with mammalian virulence were also screened. Amino acid residues were examined in the flu database [17].

### Building 3D model

The SWISS MODEL service was utilized to build the 3D models for the HA protein of F7-F10 isolates, by finding the exact templates. The PDB files were exposed by using the module of the Molsoft Internal Coordinate Mechanics (ICM-Pro) software.

## Results and discussion

**Figure 1:**
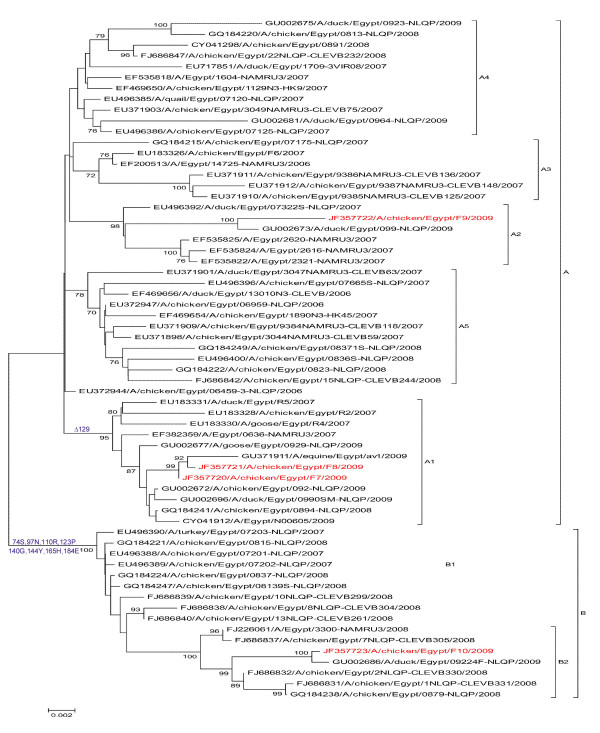
**Phylogenetic tree of viral HA sequences of the Egyptian H5N1 viruses generated by neighbour-joining analysis**. The robustness of individual nodes of the tree was assessed using a bootstrap of 1000 replications of bootstrap re-sampling of the originally-aligned nucleotide sequences. Scale bar represents 0.002 nucleotide substitutions. Viruses isolated in the current study are in red colours.

**Figure 2:**
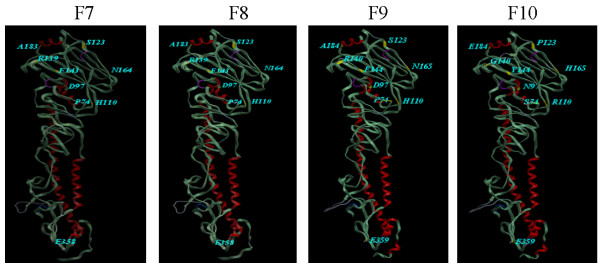
**Ribbon diagram of the trimeric HA molecule with depicts the amino acid residues differentiating variant strain (F10) from parent ones (F7-F9) shown in yellow; (P74S, D97N, H110R, S123P, R140G, F144Y, N165H, A184E)**. The majority of these sites are exterior residues. E359 (HA2- 29E) is present in F7-F10 isolates.

In the current study, four H5N1 strains were isolated from infected poultry broiler farms suffering from respiratory distress and variable mortalities; 2 from northern Egypt (Ismalia [F9] and Qaluobiya [F10]) and 2 from Middle Egypt (Fayoum Governorate [F7] and [F8]). The strains were isolated from pool organ samples that were inoculated in the SPF-ECE. All embryos died 24-48 hrs post inoculation. The infected allantoic fluid showed 3-4 haemagglutination log_2 _titers when tested with chicken RBC. The allantoic fluids reacted positively with chromatographic group and H5 antigen detecting strips. The RT-PCR for both HA and NA genes of H5N1 were found positive (data not shown). The present phylogenetic analysis, based on the analysis of the HA gene, showed that the cumulative genetic drifts in the HA gene clustered Egyptian isolates into two main lineages (A[A1-A5] and B[B1-B5]) (Figure 1). F7, A/chicken/Egypt/F7/2009 and F8, A/chicken/Egypt/F8/2009 were found to be related to lineage A1; F9 (A/chicken/Egypt/F9/2009) to lineage A2; and F10 (A/chicken/Egypt/F10/2009) to lineage B2 (Figure 1). Eight amino acid substitutions were found in the variant F10 (Figure 2) and other Egyptian variants in lineage B at the amino acid positions P74S, D 97N, H110R (epitope A), S123P, R140G (epitope B) and antigenic site), F144Y (epitope B), N165H (glycosylation site) and A184E.

The variability of amino acid in the 5 epitopes (A-E) [16] for the isolates under investigation and other available Egyptian sequences (2006-2010) in different years were screened. Residues, 43 (E epitope), 71 (E epitope), 110 (A epitope), 129 (A epitope and receptor binding site), 140 (B epitope), 141 (B epitope), 144 (B epitope), 151 (D epitope), 154 (D epitope, and glycosylation site), 185 (D epitope), 192 (D epitope) and 226 (D epitope) were subjected to high mutational pressure (Table 2).

**Table 2:** Comparative representation of the distribution of haemagglutinin epitopes that showed variation among avian and human H5N1 Egyptian isolates

Strain*	A EPITOPE														
	
	110	115	117	119	121	122	124	126	127	129	130	136	152		
F7	H	Q	I	K	S	W	D	E	A	Δ	G	P	K		
F8	H	Q	I	K	S	W	D	E	A	Δ	G	P	K		
F9	H	Q	I	K	S	W	D	E	A	S	G	P	K		
F10	R	Q	I	K	S	W	D	E	A	L	G	P	K		
2006	H	Q	I	K	S	W	D^36^/N^1^	E	A	S	G	P	K		
2007	H^71^/R^7^	Q^77^/K^1^	I^76^/F^2^	K^72^/N^6^	S^77^/C^1^	W	D^63^/N^14^/H^1^	E	A^75^/T^3^	S^66^/L^6^/Δ^6^	G	P	K		
2008	H^32^/R^50^	Q^81^/H^1^	I^75^/F^7^	K^80^/R^2^	S^80^/P^1^/Y^1^	W	D^67^/N^15^	E	A	S^66^/L^16^	G^81^/R^1^	P^77^/Q^3^/S^2^	K		
2009	H^50^/R^24^	Q^69^/K^5^	I^67^/F^4^/T^3^	R^73^/S^1^	S^73^/Y^1^	W	D^72^/N^2^	E	A	Δ^36^/L^22^/S^16^	G	P^72^/Q^2^	K^70^/E^4^		
2010	H^27^/R^17^	Q^34^/K^10^	I^43^/T^1^	K	S	W^43^/C^1^	D^42^/N^2^	E^43^/G^1^	A	Δ^26^/L^18^	G	P	K^39^/E^4^/G^1^		
Human	H^93^/R^1^	Q	I	K	S	W	D^88^/N^6^	E^93^/G^1^	A^93^/T^1^	Δ^50^/L^1^/S^43^	G	P	K^92^/Q^2^		

Strain	B EPITOPE									C EPITOPE					
		
	139	140		141	144	147	174		178	181	30		257	263	278
		
F7	G	R		S	F	V	V		I	P	Q		V	T	C
F8	G	R		S	F	V	V		I	P	Q		V	T	C
F9	G	R		S	F	V	V		I	P	Q		V	T	C
F10	G	G		P	Y	V	V		I	P	Q		V	T	C
2006	G	G		S^35^/A^2^	F	V	V		I	P	Q		V	T	C
2007	G	R^59^/K^8^/G^7 ^I^3^/S^1^		S^64^/P^14^	F^71^/Y^7^	V^76^/M^2^	V^77^/I^1^		I	P	Q		V	T	C
2008	G	G^47^/R^34^/E^1^		P^49^/S^30^/T^2^/L^1^	F^32^/Y^50^	V^80^/M^2^	V^80^/I^2^		I^81^/V^1^	P^81^/T^1^	Q^80^/K^2^		V	T^81^/A^1^	C
2009	G	R^51^/G^20^/E^1 ^I^1^/S^1^		S^46^/P^23^/T^2^/L^3^	F^51^/Y^23^	V	V^69^/I^5^		I	P^73^/S^1^	Q		V^73^/I^1^	T	C^73^/F^1^
2010	G^43^/R^1^	S^28^/P^10^/L^5^/T^1^		S^28^/P^10^/L^5^/T^1^	F^28^/Y^16^	V	V^42^/I^2^		I	P	Q		V	T	C
Human	G	R^91^/G^2^/K^1^		S^91^/P^3^	F^93^/Y^1^	V	V^92^/I^2^		I	P	Q		V	T	C

	EPITOPE D														
	
	86	101	105	151	154	155	156	158	159	161	163		185	192	

F7	A	L	L	T	D	N	A	P	T	K	S		T	Q	
F8	A	L	L	T	D	N	A	P	T	K	S		T	Q	
F9	A	L	L	I	N	N	A	P	T	K	S		A	H	
F10	A	L	L	I	N	N	T	P	T	K	S		E	K	
2006	A	L	L	I	D^34^/N^3^	N	A	P	T	K	S		A	Q	
2007	A^77^/V^1^	L^77^/M^1^	L^77^/F^1^	T^6^/I^72^	D^65^/N^10^/G^2^/B^1^	N	A^77^/V^1^	P	T	K	S		A	Q	
2008	A^79^/S^2^/V^1^	L	L^81^/S^1^	T^4^/I^77^/V^1^	D^62^/N^16^/G^3^/E^1^	N^81^/T^1^	A^66^/T^16^	P	T	K^76^/E^5^/T^1^	S^77^/N^2^/T^2^/R^1^		A^72^/E^7^/T^3^	Q^68^/K^14^	
2009	L^69^/V^5^	L	L	T^36^/I^30^/L^4^/V^4^	D^43^/N^30^/G^1^	N^71^/S^2^/D^1^	A^51^/T^23^	P	T^69^/L^3^/I^2^	K^73^/R^1^	S^73^/N^1^		A^52^/E^20^/T^2^	Q^47^/K^21^/H^6^	
2010	L^42^/S^1^/T^1^	L^43^/M^1^	L	T^28^/I^16^	N^32^/D^12^	N^43^/D^1^	A^27^/T^17^	P^43^/L^1^	T^39^/L^5^	K	S^39^/N^5^		A^28^/E^12^/T^3^/V^1^	Q^27^/K^17^	
Human	A^90^/S^2^/T^2^	L	L	T^50^/I^44^	D^61^/N^29^/G^3^/E^1^	N^93^/S^1^	A^93^/T^1^	P	T	K	S		A^88^/E^3^/T^3^	Q^89^/H^4^/K^1^	

Strain	EPITOPE D									EPITOPE E					
		
	197	198	199	210	212	226	43	47	66	71	72	75	83	244	249
		
F7	Y	I	S	V	K	M	N	V	M	L	N	E	I	N	A
F8	Y	I	S	V	K	M	N	V	M	L	N	E	I	N	A
F9	Y	I	S	V	K	M	D	V	M	L	N	E	I	N	A
F10	Y	I	S	V	K	V	D	V	M	P	N	E	I	N	A
2006	Y	I	S	V	K	M^35^/I^2^	D	V	M	L	N	E	I	N	A
2007	Y^77^/Q^1^	I	S	V	K	M^46^/V^7^/I^25^	D^72^/N^6^	V^77^/M^1^	M^77^/L^1^	L^77^/H^1^	N^72^/D^6^	E	I^77^/V^1^	N	A
2008	Y	I	S^80^/P^1^	V^76^/I^6^	K^80^/T^1^	M^23^/V^49^/I^10^	D^78^/N^4^	V	M	L^73^/P^5^/F^4^	N	E^81^/K^1^	I	N	A
2009	Y	I	S	V	K^70^/R^4^	M^43^/V^23^/I^8^	D^38^/N^36^	V	M	L^55^/P^19^	N	E	I^68^/T^4^/S^2^	N^73^/D^1^	A
2010	Y	I^43^/V^1^	S	V^43^/A^1^	K	M^27^/V^17^	D^17^/N^26^/S^1^	V	M	L^32^/P^12^	N	E	I^39^/T^5^	N	A^43^/V^1^
Human	Y	I	S	V^93^/I^1^	K^93^/Q^1^	M^90^/V^1^/I^3^	D^44^/N^50^	V^93^/M^1^	M	L	N	E	I	N	A

*: Egyptian; avian strain from tested (F7-F10) and published sequences in the flu database from 2006-2010 as well as Egyptian human published sequences (2006-2010).

Epitopes allocation were determined previously by Duvvuri et al [16].

Δ: Deletion,

Superscripts denote the number of isolates that possess the specified amino acid residue.

Avian isolates: 2006: n=37, 2007: n=78, 2008: n=82, 2009:n=74, 2010:n=44   Human isolates: n=94.

F7 and F8 viruses and other viruses in the A1 group showed a conspicuous deletion of amino acid 129 in the HA protein (H5 numbering) (Table 2). The amino acid residue number 129 (S in F9 and L in F10) is located in the epitope A (Table 2) and part of the receptor binding site and also belongs to an antigenic site [16]. The 129 deletion was not present in the ancestor A/Goose/Guangdong/1/96 strain [18] or in the H5N1 viruses originally introduced into Egypt in 2006. S129L substitution and 129 deletion were detected in 2007 (L in 6/78 isolates and deletion in 6/78 isolates), 2008 (L in 16/82, deletion in 0/82), 2009(deletion in 36/74, L 22/74) and 2010 (deletion in 26/44 and L in 18/44) (Table 2). Serine at site 129 is a receptor-binding site for α-2,3-linked sialic acids. Previously, it was reported that a mutation at this site (S129L) decreased virulence of HPAIV H5N1 in mice [19]. However, a structural study on the H5N1 HA has reported that change from serine to leucine facilitates access to α-2,6-linked sialic acids [20]. The 129S deletion was not detected in the 2006 human isolates (0/16), however, in 2007-2010, the 129 deletion was detected in (5/24) of 2007, (2/6) of 2008, (31/34) of 2009 and (12/12) 2010 human cases. Isolates from nonhuman mammals (equine, n = 1) like those in the majority of human in 2009 and 2010 possessed the 129-deletion. Interestingly, there was a similar deletion in the HA protein at the corresponding position of all human seasonal H1N1 and H3N2 viruses. It should be noted that this position is close to a domain modulating receptor interaction. Likewise, it is also interesting that strains with this deletion seem to evolve towards a receptor usage that is similar to human H1N1[21].

The deduced amino acid exchanges, as with most H5N1 Egyptian strains, are distributed across the HA protein and are found in the polybasic cleavage motif, whose consensus sequence is PGERRRKKR/GLF for clade 2.2 viruses. All the isolated strains possessed a multibasic HA0 amino acid cleavage sequence characteristic for the highly pathogenic H5N1 strains clade 2.2. While the sequence diversity of the haemagglutinin open reading frame of F7 and F8 had cleavage site of the consensus of 2.2, F10 PQGEGRRKKR/GLF showed (R325G) substitution and F9 showed two amino acid substitutions in the cleavage site PQGKSRRKKR/GLF, (E324K and R325S). All variations within the polybasic cleavage motif probably do not affect cleavage of the HA precursor molecule since F7-F10 have the RX(K/R)R consensus motif, for the cleavage by furin or the subtilisin-like protein convertases [22] which remains conserved. The significance of the change in the cleavage site, however, remains to be understood.

The HA sequences contained different N-linked potential glycosylation sites, F10 had eight potential sites at the following positions: 11, 23, 72, 236, 273, 286, 484 and 543. F7 and F8 possess 6 sites, at 11, 23, 164, 285, 483, 542 whereas F9 possessed only 5 sites at 23, 165, 286, 484, 543. F10 acquired two additional glycosylation sites (72 [epitope E] and 236 [polybasic cleavage site]) but lost a glycosylation site at165 that was located near the epitope D. F9 lost the glycosylation site at 11 position (H5 Numbering).

Lycett et al. specified 6 amino acid residues (86V, 124S, L/N138, T/S156, E/R212, T263) that are linked to the virulence of H5N1 in mammals [23]. T156 and T263 were also present in F10. V86 was also present in 1/78 (2007), 1/82 (2008),5/74 (2009) isolates but none of the human isolates. T156 was present in 16/82, 23/74 and 17/44 of avian isolates in 2008, 2009 and 2010 isolates respectively, however it was present in 1/94 human isolates (A/Egypt/3300-NAMRU3/2008). T263 was present in F7-F10 isolates in addition to all human and 314/315 avian isolates (Table 3). On the other hand, while S129L and A156T substitutions were found to be associated with viral adaptation to poultry [24], both substitutions were found in F10 isolate.

**Table 3:** Amino acid sites associated with virulence to mammals in Egyptian avian and human isolates

H5 Numbering			Egyptian Isolates									
			
	Residue	Human n = 94	Isolates in the current study				Sequences of Egyptian avian isolates in the GenBank					Reference
				
			F7	F8	F9	F10	2006	2007	2008	2009	2010	
							n = 37	n = 78	n = 82	n = 74	n = 44	
86	V^v^/A, I, P, S, T	A^90^/S^2^/T^2^	2A	A	A	A	A	A^77^/V^1^	A^79^/S^2^/V^1^	A^69^/V^5^	A^42^/S^1^/T^1^	[32]
124	S^v^/N, D	D^88^/N^6^	D	D	D	D	D ^36^/N ^1^	D^63^/N^14^/H^1^	D^67^/N^15^	D^72^/N^2^	D^42^/N^2^	[32]
138	L^v^, N^v^/Q, H, I	Q	Q	Q	Q	Q	Y	Y^72^/H^6^	Q^80^/H^1^/K^1^	Q^72^/H^2^	Q^42^/H^1^/K^1^	[32]
156	T^v^/S^v^/A	A^93^/T^1^	A	A	A	T	A	A^77^/V^1^	A^66^/T^16^	A^51^/T^23^	A^27^/T^17^	[32-34]
212	E^v^/R^v^/K	K^93^/Q^1^	K	K	K	K	K	K	K^81^/T^1^	K^70^/R^4^	K	[32]
263	T^v^/A	T	T	T	T	T	T	T	T^81^/A^1^	T	T	[32]

Numerical superscripts denote the number of isolates that possess the specified amino acid residue.

V superscript denotes a residue that has been found to be associated with an increase in the virulence of H5N1 to mammals.

The K189R amino acid substitution at the receptor-binding site was reported as an escape mutant change [25] and was further clearly shown to be significantly antigenic [19]. Only, 4/315 isolates of the Egyptian GenBank sequences showed K189 while K189R was present in the F7-F10 isolates as well as 92/94 human and 296/315 avian Egyptian strains. Accordingly, it does not seem to be linked to escape mutants in the Egyptian strains. Duvvuri et al., analyzed potential mutational changes in L138Q, R140K and A156T [16]. The amino acids at sites 138 and 140 were involved in the carbohydrate specificity of HA molecule towards host receptor [26]. L138Q was present in the F7-F10, 80/82 of 2008 isolates, 72/74 of 2009 isolates and 42/44 of 2010 isolates and all human strains. On the other hand, R140K substation was present in only 8/78 of 2007 avian isolates but none of the human strains.

HA2 29E (H5 numbering 359) was detected in the F7-F10 isolates (Figure 2) and in all Egyptian human and avian isolates other than the A/chicken/Egypt/34-2/2008 and the A/chicken/Egypt/3046NAMRU3-CLEVB62/2007. All viruses in clade 1 and 2 possessed E29 in the HA2 subunit. The K29E substitution resulted in loss of a proteasomal cleavage site. The Glu-29 in the HA2 subunit may have been helpful to H5N1 viruses since such change induced the loss of one proteasomal cleavage site, which means that the virus can provoke a lower cytotoxic T cell response [27]. All human and avian strains possessed E359 with the exception of two isolates; A/chicken/Egypt/3046NAMRU3-CLEVB62/2007 and A/chicken/Egypt/34-2/2008, which possessed K359 and N359 respectively.

## Conclusions

Egypt is a good candidate of the study of AIV genetic evolution under high vaccination pressure as there is a high avian population density, significant inter-species and intra-species transmission and as the vaccination has been a regular veterinary practice since 2006. We identified several important differences in HA gene from the 4 H5N1 strains under investigation and the available Egyptian H5N1 sequences in the flu database and specified amino acids substitutions that: distinguished variant from classical parent strains, those linked to the virulence of H5N1 in mammals, and those present in different HA epitopes. The HA of the Egyptian H5N1 strains was found to undergo intensive genetic changes in a complex pattern. It has been speculated that the avian influenza vaccination has resulted in accelerated genetic drifts in the HA gene sequence [28]. Nevertheless, other researchers have suggested that viral replication, *per se*, allows for the expression of drifts among subsequent viral populations [29-31]. The presence of mutant strain, that could replicate and shed in spite of vaccination and possesses amino acid substitutions linked to virulence in mammals, necessitates the importance of employing more rigorous requisites than those being utilized by the World Organization of Animal Health (OIE) which only considers protection afforded by the vaccine in terms of bird survival rates.

## Competing interests

The authors declare that they have no competing interests.

## Authors' contributions

ASA carried out all the experiments including designing the experiments, acquisition of data, analysis and interpretation of data. and drafted the manuscript. MAA and MFE and have helped in acquisition of samples, data analysis and revising the manuscript. All authors have read and approved the final manuscript.
